# MicroRNA-21 increases cell viability and suppresses cellular apoptosis in non-small cell lung cancer by regulating the PI3K/Akt signaling pathway

**DOI:** 10.3892/mmr.2020.11758

**Published:** 2020-12-04

**Authors:** Tao Wang, Zhenyu Cai, Guolin Hong, Gangsen Zheng, Yu Huang, Shun Zhang, Jinhua Dai

Mol Med Rep 16: 6506-6511, 2017; DOI: 10.3892/mmr.2017.7440

Following the publication of this paper, the authors have contacted the Editorial Office to request that their article be retracted. The reason for this retraction is an inability to be able to replicate certain of their previous results, and also disagreements among the authors as to the interpretation of some of the data. Following further discussion, all authors and the Editor of *Molecular Medicine Reports* are in agreement that the paper should be retracted; moreover, the authors apologize to the readership for any inconvenience caused.

